# *Jnk2* deficiency increases the rate of glaucomatous neurodegeneration in ocular hypertensive DBA/2J mice

**DOI:** 10.1038/s41419-018-0705-8

**Published:** 2018-06-13

**Authors:** Jeffrey M. Harder, Pete A. Williams, Ileana Soto, Nicole E. Foxworth, Kimberly A. Fernandes, Nelson F. Freeburg, Richard T. Libby, Simon. W. M. John

**Affiliations:** 1 The Jackson Laboratory, Bar Harbor, ME 04609 USA; 2 Molecular and Cellular Biosciences Department, Rowan University, Glassboro, NJ 08028 USA; 3 Flaum Eye Institute, Department of Ophthalmology, University of Rochester Medical Center, Rochester, NY 14642 USA; 4 The Howard Hughes Medical Institute, The Jackson Laboratory, Bar Harbor, ME 04609 USA; 5 Department of Ophthalmology, Tufts University School of Medicine, Boston, MA 02111 USA

## Abstract

The cJun N-terminal kinases (JNKs; JNK1, JNK2, and JNK3) promote degenerative processes after neuronal injury and in disease. JNK2 and JNK3 have been shown to promote retinal ganglion cell (RGC) death after optic nerve injury. In their absence, long-term survival of RGC somas is significantly increased after mechanical optic nerve injury. In glaucoma, because optic nerve damage is thought to be a major cause of RGC death, JNKs are an important potential target for therapeutic intervention. To assess the role of JNK2 and JNK3 in an ocular hypertensive model of glaucoma, null alleles of *Jnk2* and *Jnk3* were backcrossed into the DBA/2J (D2) mouse. JNK activation occurred in RGCs following increased intraocular pressure in D2 mice. However, deficiency of both *Jnk2* and *Jnk3* together did not lessen optic nerve damage or RGC death. These results differentiate the molecular pathways controlling cell death in ocular hypertensive glaucoma compared with mechanical optic nerve injury. It is further shown that JUN, a pro-death component of the JNK pathway in RGCs, can be activated in glaucoma in the absence of JNK2 and JNK3. This implicates JNK1 in glaucomatous RGC death. Unexpectedly, at younger ages, *Jnk2*-deficient mice were more likely to develop features of glaucomatous neurodegeneration than D2 mice expressing *Jnk2*. This appears to be due to a neuroprotective effect of JNK2 and not due to a change in intraocular pressure. The *Jnk2-*deficient context also unmasked a lesser role for *Jnk3* in glaucoma. *Jnk2* and *Jnk3* double knockout mice had a modestly increased risk of neurodegeneration compared with mice only deficient in *Jnk2*. Overall, these findings are consistent with pleiotropic effects of JNK isoforms in glaucoma and suggest caution is warranted when using JNK inhibitors to treat chronic neurodegenerative conditions.

## Introduction

Glaucoma is a common neurodegenerative disease that causes vision loss, affecting millions of people worldwide^1^. Glaucomatous degeneration includes loss of retinal ganglion cell (RGC) somas in the retina and loss of their axons, which extend from the retina into the optic nerve and brain. In glaucoma, RGC axons show early signs of damage in the lamina, the region where RGC axons exit the eye^2–6^. Damage at this site is important because it may trigger the molecularly distinct degeneration processes that occur on each side of this site^7–10^. In the DBA/2J (D2) mouse model of glaucoma, genetically disrupting *Bax* prevents loss of RGC somas (RGC death) and their axons up to the lamina; however, it does not stop degeneration of the optic nerve outside of the eye and distal to this site of injury^8^. Furthermore, the expression of *Wld*^*S*^, a mutation that protects against axonal degeneration, can curb early damage to RGC axons in the lamina^11^ and protect RGCs and the optic nerve^4,11^. Thus, identifying the molecular mechanism(s) that leads to progressive axon damage in the lamina is an important step toward developing neuroprotective glaucoma treatments.

The cJun N-terminal kinase (JNK) signaling pathway regulates how various neurons respond to axon damage^12^. In RGCs, severe axon trauma activates a specific subset of JNK signaling pathway members ahead of cell death. Consistent with specific JNK signaling pathway members causing RGC death, disrupting the upstream kinase dual leucine zipper kinase (DLK encoded by *Map3k12*), or the downstream JNK target JUN, lessens RGC death after mechanical optic nerve injury^13–18^. JNK itself is a product of three distinct mitogen-activated protein kinase genes (*Mapk8*, *Mapk9*, and *Mapk10*, encoding JNK1, JNK2, and JNK3, respectively, and referred to as *Jnk1, Jnk2*, and *Jnk3* in this article), all of which are expressed in the nervous system^19^. DLK and JUN are linked in the axon injury-induced RGC death pathway by JNK2 and JNK3^14,17^. The combined absence of JNK2 and JNK3 prevents JUN activation and RGC death after mechanical optic nerve injury similar to deficiencies in *Map3k12* or *Jun*. It is hypothesized that a similar cell death pathway is activated by axon damage in the lamina that occurs in glaucoma. *Jun* deficiency has been recently shown to lessen RGC death in D2 mice with glaucoma^15^ and other molecules in the JNK signaling pathway are implicated in glaucoma as well^20–22^. Thus, to better understand the function of JNKs in glaucoma, we tested the function of JNK2 and JNK3 in DBA/2J mice, an ocular hypertensive model of glaucoma^23–26^.

## Results

### IOP elevation activates JNK signaling in the retina and optic nerve

**Fig. 1:**
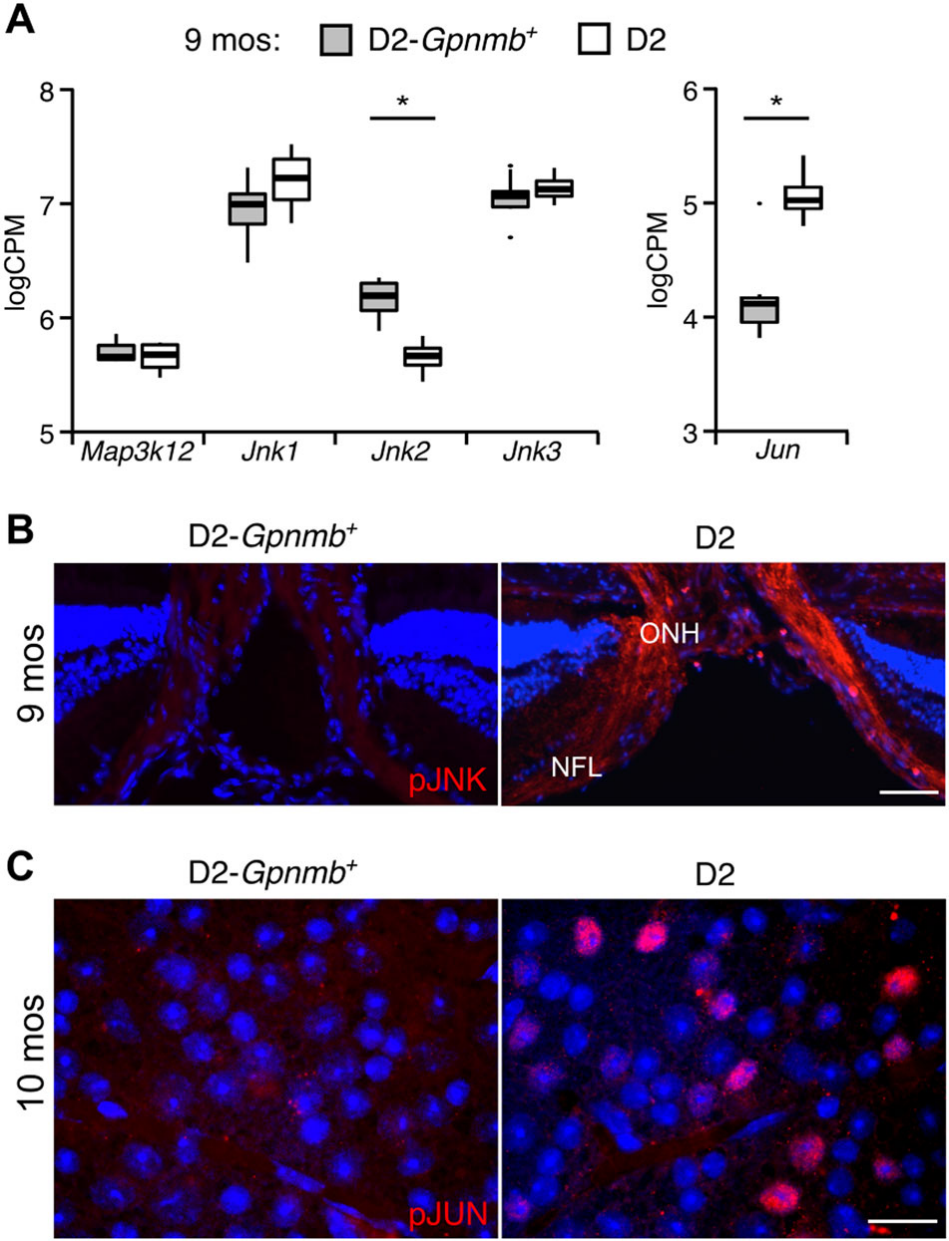
JNK pathway genes associated with axon injury in RGCs are expressed and activated during D2 glaucoma at 9 months of age. **a** Publically available RNA-sequencing data sets from RGCs were assessed for expression of a subset of JNK signaling pathway genes implicated in axon injury. RGCs from a group of D2 eyes with ocular hypertension, but not optic nerve damage were compared with RGCs from control D2-*Gpnmb*^*+*^ eyes that do not have high intraocular pressure or develop glaucoma. The D2 eyes analyzed here belong to the group of eyes with the earliest detected changes in this model (Group 2,^29^). The Tukey-style box plots were generated in R. The thick bar represents median expression level and the hinges correspond to the first and third quartiles. Whiskers represent 1.5 × the interquartile range, with outlying data points plotted individually. Expression level is shown as log counts per million (log_2_CPM). Significant differences in expression of *Jnk2* and *Jun* were detected. **b** Phosphorylated S63 pan-JNK immunoreactivity (pJNK, red) in ocular hypertensive (D2) and normotensive (D2-*Gpnmb*^*+*^) eyes was assessed by fluorescence in cross-sections that include the retina and the optic nerve head (ONH). The nerve fiber layer (NFL) is filled by RGC axons as they extend toward the ONH. DAPI (blue) labeling of nuclei was used as a counterstain. **c** Phosphorylated JUN immunoreactivity (pJUN, red) in the ganglion cell layer was assessed by fluorescence in retinal flat mounts with DAPI (blue) used as a counterstain. Representative images from ocular hypertensive (D2) and normotensive (D2-*Gpnmb*^*+*^) eyes are shown. *FDR < 0.05. Scale bar: 50 μm **b**, 25 μm **c**

Using D2 and D2-*Gpnmb*^*+*^ mice, we assessed whether RGCs expressed JNK signaling pathway genes during the window of ocular hypertension in D2 mice. By 9 months of age (mos), most D2 eyes have ongoing periods of elevated intraocular pressure (IOP), but typically no optic nerve damage or RGC death^25^. At this age, many eyes have gene expression differences compared with age-matched D2-*Gpnmb*^*+*^ eyes that do not develop high IOP and glaucoma^4,27–30^. Previously, RNA-seq-based transcriptomes of RGCs purified from D2 and D2-*Gpnmb*^*+*^ eyes at 9 mos of age were assembled^29^. In ocular hypertensive eyes, RGCs express a number of JNK signaling pathway genes previously implicated in the axon injury-induced RGC death pathway (Fig. 1a). A significant difference in the expression of *Jnk2* and *Jun* exists between D2 and D2-*Gpnmb*^*+*^ RGCs (false discovery rate (FDR) < 0.05).

To further examine JNK signaling in glaucoma, we determined if JNK and JUN were phosphorylated in the retina using immunohistochemistry (pJNK and pJUN, respectively, phosphorylation induces an activated state). At 9 mos of age, pJNK was present in the nerve fiber layer and optic nerve head (ONH, Fig. 1b) in D2 eyes, but was undetectable in control D2.*Gpnmb*^*+*^ eyes. pJUN-positive cells were detectable throughout the ganglion cell layer at 10 mos in D2 eyes, but not in D2.*Gpnmb*^*+*^ eyes (Fig. 1c). These data are consistent with recent findings, which showed pJUN in TUJ1 + RGCs in glaucomatous DBA/2J mice^15^. Together, these data indicate that the JNK signaling pathway responds to elevated IOP with changes in both expression level and activation state of key molecules.

### *Jnk2* and *Jnk3* deficiencies do not prevent pigment disease or IOP elevation in DBA/2J mice

**Fig. 2:**
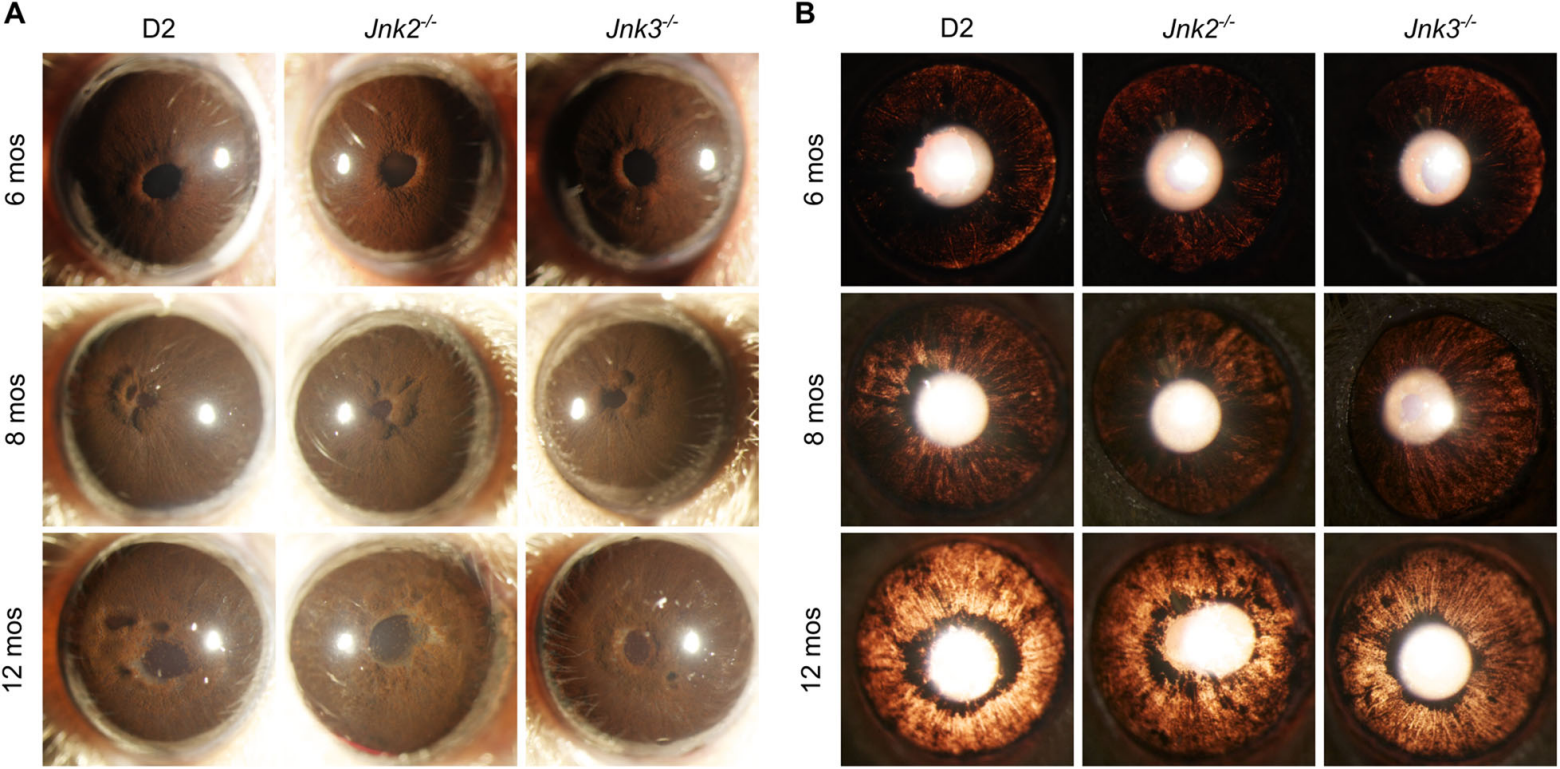
*Jnk* genotype has no affect on iris disease. Images of eyes were taken using a slit lamp biomicroscope. **a** Representative broad beam images demonstrating similar iris abnormalities in mice of all genotypes. **b** Representative images demonstrating the similar transillumination defects resulting from iris depigmentation in D2, D2.*Jnk2*^*−/−*^, and D2.*Jnk3*^*−/−*^ mice

**Fig. 3:**
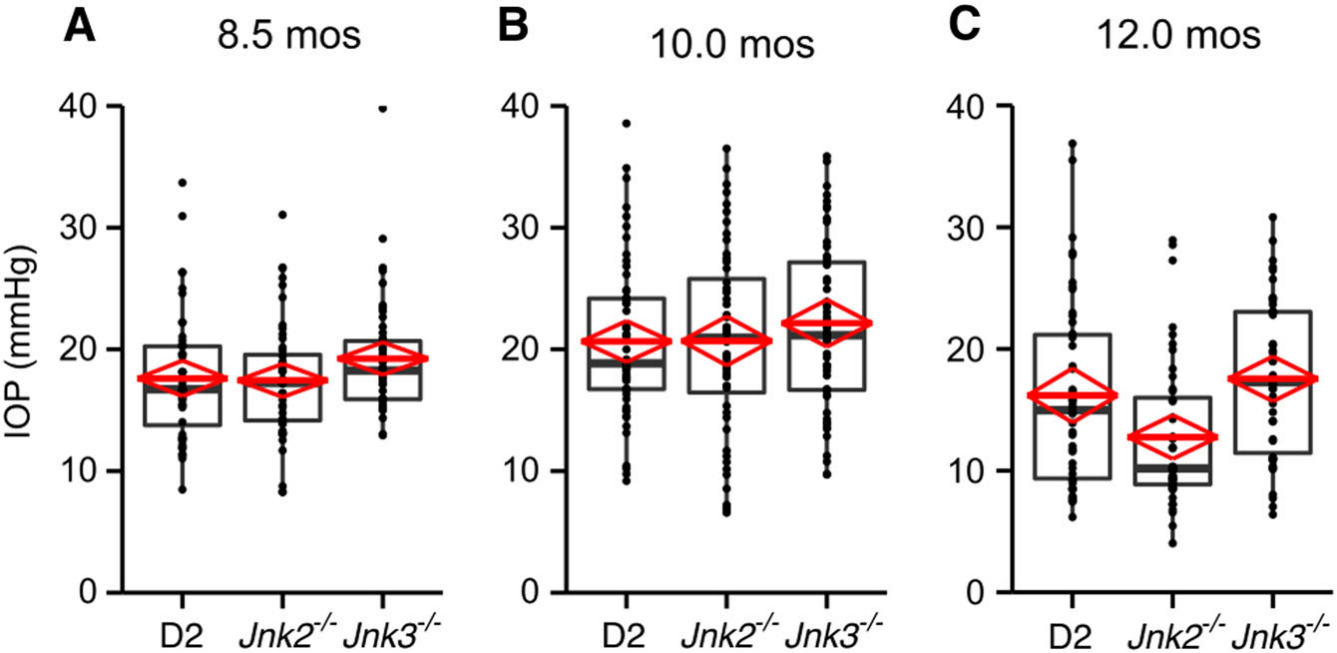
Deficiency of *Jnk2* or *Jnk3* does not alter intraocular pressure profiles in D2 mice. IOP elevation occurs in *Jnk2* and *Jnk3*-deficient mice at 8.5 mos **a**, 10.0 mos **b**, and 12.0 mos **c**. IOP elevations sufficient to cause glaucoma were observed in mice of all genotypes. No significant differences were found when comparing *Jnk*-deficient with control D2 mice (*n* = 40 per group, *P* *>* 0.05 in each comparison. However, at 12 mos *Jnk2*-deficient mice tended to have lower IOP than controls (*n* = 42, *P* = 0.052). IOP levels start to decline in D2 mice at 12 mos due to ciliary body degeneration and decreased production of aqueous humor. These data do not exclude a role for JNK2 in lessening this late disease process in the ciliary body, but this IOP difference may also be due to chance variation. In the boxplot, the boxes define the 25th and 75th percentiles. The black line in each box represents the median value. The red diamonds indicate the mean value and the 95% CI. The full range of the data points is also depicted

Ocular hypertension in D2 mice occurs secondary to a pigment dispersing iris disease^23,25^. D2 mice carrying null alleles of *Jnk2* and *Jnk3* were used to test the role of JNK2 and JNK3 in glaucoma and were maintained in a single mouse colony. As no haploinsufficiency induced phenotypes have been reported for *Jnk2* and *Jnk3*, D2 mice with one or more functional copies (^*+/+*^ or ^+/−^) of both *Jnk2* and *Jnk3* were grouped together as control mice in this study. D2.*Jnk2*^*−/−*^ mice were either wild-type or heterozygous for *Jnk3*. Similarly, D2.*Jnk3*^*−/−*^ mice were either wild-type or heterozygous for *Jnk2*. Eyes of all genotypes were carefully assessed for the iris disease and IOP elevation. No differences in iris disease occurred between genotypes (Fig. 2). The iris disease in eyes of all genotypes (*n* = 40 eyes/group) was readily apparent between 6 and 7 mos and most eyes had severe iris disease by 12 mos. Consistent with the iris disease, ocular hypertension occurred in mice of all genotypes as measured between 8 and 12 mos (Fig. 3). In all groups of mice, IOP distributions were closely matched with a similar range of IOP levels until 12 mos, an age when levels of IOP start to decrease in this model^25^. Declining IOP was most evident in D2.*Jnk2*^*−/−*^ mice compared with controls; however, this difference was not significant (*P* = 0.052). Overall, these data indicate that deletion of *Jnk2* or *Jnk3* does not alter the iris disease and IOP insult in D2 mice.

### *Jnk2* deficiency increases susceptibility to glaucomatous optic nerve damage

**Fig. 4:**
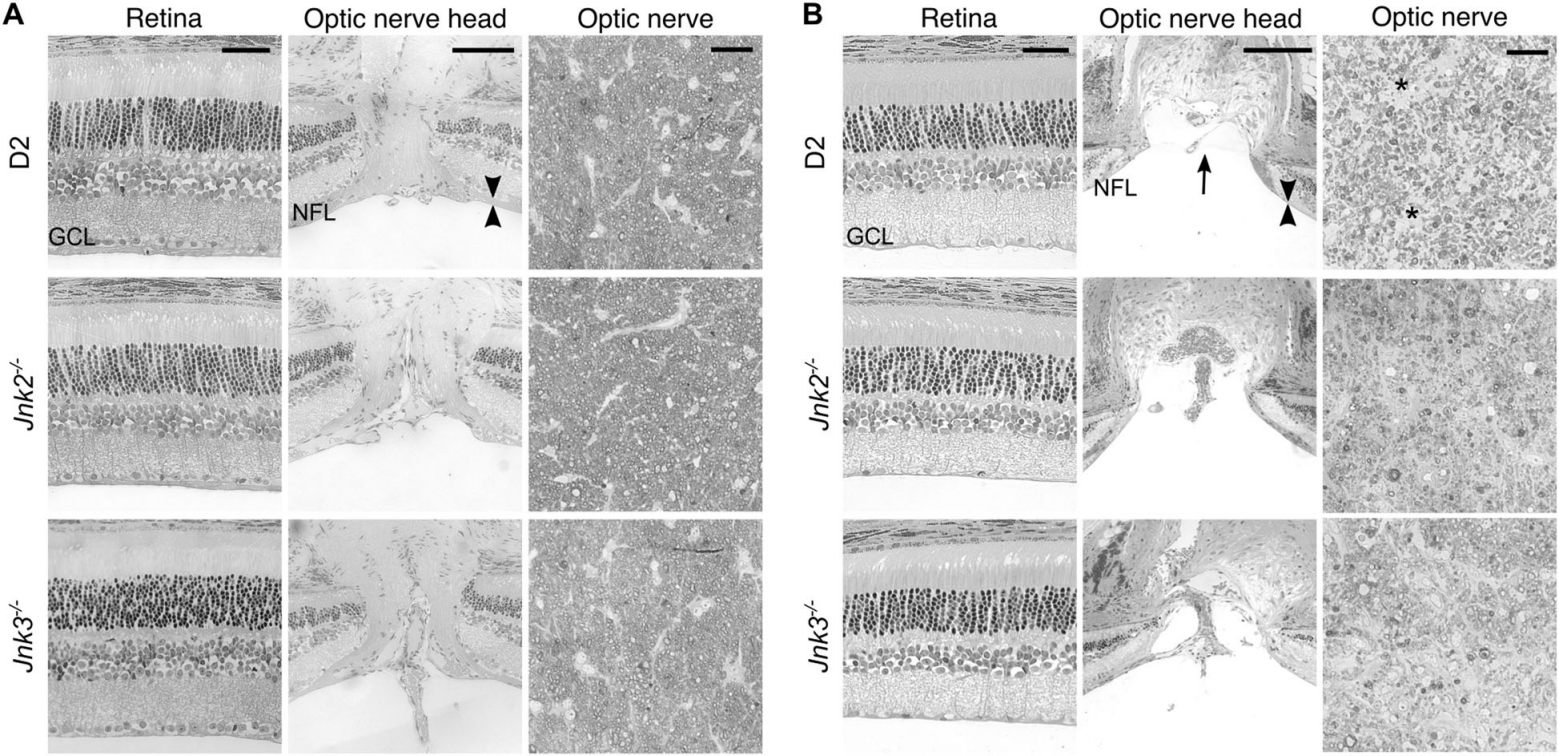
Histological assessment of retina and optic nerve. Representative images from H&E-stained ocular cross-sections and PPD stained optic nerves are shown for unaffected eyes (6 months of age) **a**, and severely affected eyes (12 months of age) **b**. These images demonstrate the hallmark degenerative features of glaucoma including RGC layer (GCL) neuron loss in the retina, nerve fiber layer thinning (compare arrowheads in  **a** and **b**), optic disc cupping (arrow), and sick and degenerating axons (darkly stained dots), axon loss and glial scarring (asterisks) in the optic nerve. Scale bars: 50 μm (retina), 100 μm (optic nerve head), 25 μm (optic nerve)

Key degenerative features of glaucoma include selective loss of RGCs, cupping of the optic disc, and degeneration of the optic nerve. Elevated IOP in wild-type D2 mice (D2. *Jnk2*^*+/+*^*Jnk3*^*+/+*^) leads to a well-characterized glaucoma with these key features^25^. To determine whether D2.*Jnk2*^*−/−*^ and D2.*Jnk3*^*−/−*^ mice developed glaucoma, the gross anatomy of their retinas and optic nerves were examined. At 6 mos of age, retina and ONH anatomy was normal in D2.*Jnk2*^*−/−*^ and D2.*Jnk3*^*−/−*^ eyes (*n* = 6 eyes/genotype, Fig. 4a). At 12 mos, D2.*Jnk2*^*−/−*^ and D2.*Jnk3*^*−/−*^ eyes with optic nerve degeneration all exhibited the hallmark features of glaucomatous degeneration with no obvious differences to those observed in D2 mice (*n* = 6 eyes/genotype, Fig. 4b).

**Fig. 5:**
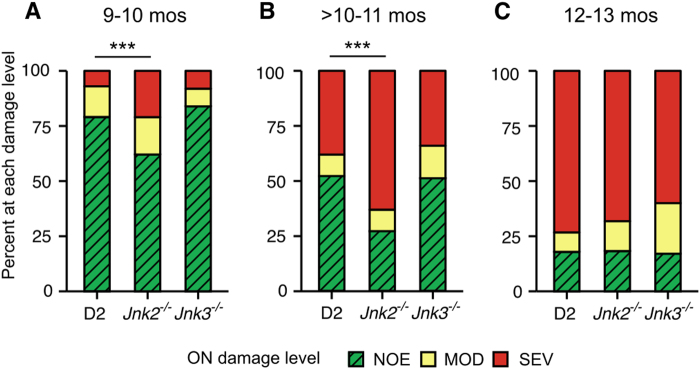
*Jnk2* deficiency increases optic nerve vulnerability to high IOP. The severity of optic nerve damage was determined using PPD stained cross-sections (see Materials and methods for details) at 9–10 mos **a**, > 10–11 mos **b**, and 12–13 mos **c**. Nerves were determined to have no glaucomatous nerve damage (no or early, NOE—no glaucoma based on nerve damage but called NOE as some of these eyes have early transcriptional changes that precede degeneration^30^), moderate disease (MOD) or severe disease (SEV). An increased number of eyes from *Jnk2* mice had glaucomatous nerve damage compared with control D2 mice at 9.5 and 10.5 mos. ***P < 1 × 10^−5^

The degree of optic nerve degeneration was determined in larger numbers of mice by analyzing optic nerve cross-sections from mice aged between 9 and 13 mos (see Materials and methods for details of damage determination). Consistent with IOP elevation, the number of eyes with optic nerve degeneration increased with age for all genotypes (Fig. 5). At each time point, there was no significant difference in the distribution of injury between optic nerves from D2.*Jnk3*^*−/−*^ and control mice. Thus, complete absence of *Jnk3*, either alone or in combination with a single, heterozygous, null allele of *Jnk2*, did not affect optic nerve degeneration. In contrast, in the 9–10 mos group significantly more D2.*Jnk2*^*−/−*^ optic nerves developed glaucomatous damage than control D2 optic nerves (39% vs. 22% respectively; *n* = 60 eyes/genotype, *P* < 0.001; Fig. 5a). The increased susceptibility was still present in 10- to 11-month group, with, 73% of D2.*Jnk2*^*−/−*^ eyes having optic nerve damage compared with 48% of control D2 eyes (*n* = 80 eyes/genotype, *P* < 0.001, Fig. 5b). In the 12- to 13-month group, a time point when the vast majority of D2 mice that will develop glaucomatous neurodegeneration have already done so (and so used as an end stage)^25^, there was no difference between genotypes (*n* = 45 eyes/genotype, Fig. 5c). Thus, the absence of *Jnk2* is associated with increased susceptibility to ocular hypertension-induced optic nerve damage (by increasing the likelihood of developing damage at earlier ages with shorter exposure to high IOP).

### Loss of *Jnk3* increases optic nerve damage in *Jnk2*^*−/−*^ mice

**Fig. 6:**
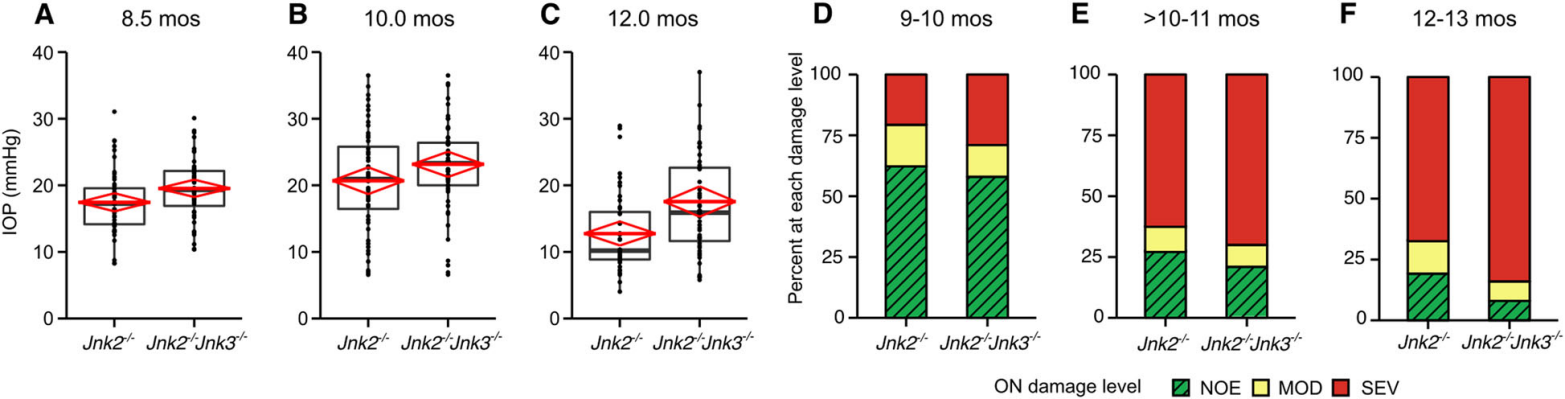
*Jnk3* deficiency subtly exacerbates glaucoma in mice lacking *Jnk2*. **a**–**c** IOP elevations sufficient to cause glaucoma occurred for both genotypes with no significant differences between genotypes at 8 and 10.5 mos. At 12 mos, the difference between *Jnk2*^*−/−*^ and *Jnk2*^*−/−*^ *Jnk3*^*−/−*^ genotypes was due to the IOP decrease observed in *Jnk2* only deficient mice (*P* = 0.042). The IOP levels in mice with combined *Jnk2* and *3* deficiencies closely resembled those of wild-type, heterozygous, and *Jnk3*-deficient mice (compare with Fig. 3c). Thus, the greater decrease in IOP in the *Jnk2*^*−/−*^ *mice* may be due to chance, as it is hard to reconcile why the additional deficiency of *Jnk3* in *Jnk2*^*−/−*^ *Jnk3*^*−/−*^ mutant mice would prevent it. **d**-**f** The severity of optic nerve damage was determined using PPD stained cross-sections (see Materials and methods for details). Data for D2.*Jnk2*^*−/−*^ mice are the same data as in Fig. 5 as mice of all genotypes were produced in the same litters and examined at the same time

Eyes of D2.*Jnk2*^*−/−*^*Jnk3*^*−/−*^ mice developed ocular hypertension and optic nerve damage consistent with glaucoma as for other genotypes (Fig. 6). IOP levels in D2.*Jnk2*^*−/−*^ and D2.*Jnk2*^*−/−*^*3*^*−/−*^ mice were closely matched until 12 mos, when IOP in more D2.*Jnk2*^*−/−*^ eyes had decreased (see above). IOPs profiles of D2.*Jnk2*^*−/−*^*3*^*−/−*^ eyes closely resembled those of wild-type, heterozygous, and D2.*Jnk3*^*−/−*^ mice (compare with Fig. 3c). The natural decline in IOP starting at 12 mos was not affected by combined *Jnk2* and *Jnk3* deficiency. The combination of *Jnk2* and *Jnk3* deficiencies had a small effect on optic nerve damage compared with *Jnk2* deficiency alone. In aggregate, between 9 and 13 mos in these age- and sex-matched groups, more D2.*Jnk2*^*−/−*^*Jnk3*^*−/−*^ eyes had a severely damaged optic nerve than D2.*Jnk2*^*−/−*^ eyes (107 *Jnk2*^*−/−*^*3*^*−/−*^ compared with 89 *Jnk2*^*−/−*^ eyes; *n* = 185 eyes/genotype, *P* = 0.007). Based on these data, both *Jnk2* and *Jnk3* appear to influence glaucoma pathogenesis in D2 mice.

### JNK2 and JNK3 are not necessary for RGC death in glaucoma

**Fig. 7:**
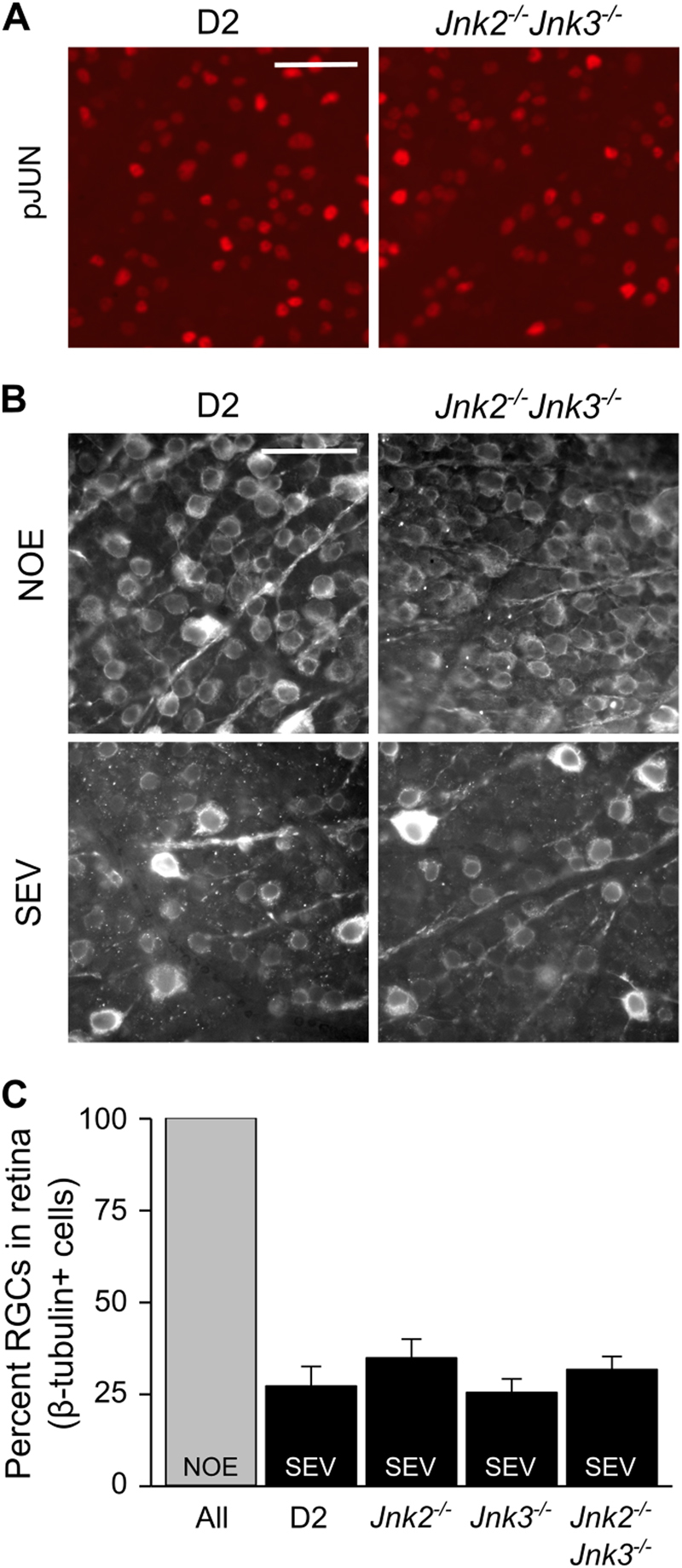
JNK2 and JNK3 are not necessary for RGC somal degeneration in glaucoma. **a** The JNK target JUN is known to promote RGC apoptosis in D2 glaucoma^15^. Double deficiency of *Jnk2* and *Jnk3* did not prevent accumulation of phosphorylated JUN (pJUN) immunoreactivity (red) at 10 mos. pJUN was detected by immunofluorescence in retinal flat mounts. **b** β-Tubulin is an RGC-specific marker in the retina and was used to label RGCs in retinal flat mounts at 12 mos. Representative images show loss of RGCs in eyes with severe optic nerve degeneration compared with healthy (NOE) eyes. Occasional RGCs have higher β-tubulin immunoreactivity (bright cells). **c** Quantification of β-tubulin-positive cells suggests that *Jnk2* and *Jnk3* deficiency did not affect somal degeneration of RGCs in eyes with severe optic nerve loss. RGC counts were similar in NOE eyes for all genotypes (represented together as All)

Recently, *Jun* deficiency was shown to lessen RGC death after both mechanical optic nerve injury and in ocular hypertensive D2 mice with optic nerve damage^13,15^. Furthermore, after mechanical optic nerve injury, JUN activation has been shown to be dependent on JNK2 and JNK3^13,15^. To determine whether *Jnk2* and *Jnk3* are required for JUN phosphorylation and RGC death in glaucoma, we assessed pJUN immunoreactivity and RGC soma loss in D2.*Jnk2*^*−/−*^*Jnk3*^*−/−*^ mice. At 10 mos, we detected pJUN in 6 out of 6 D2.*Jnk2*^*−/−*^*Jnk3*^*−/−*^ retinas and 6 out of 6 control D2 retinas examined (Fig. 7a). At 12–13 mos, RGC soma loss as assessed by counts of β-tubulin-positive RGCs was observed in 5 out of 5 D2.*Jnk2*^*−/−*^*Jnk3*^*−/−*^ eyes with severe optic nerve damage (Fig. 7b). Similar loss of RGC somas was observed in D2, D2.*Jnk2*^*−/−*^, D2.*Jnk3*^*−/−*^, and D2.*Jnk2*^*−/−*^*Jnk3*^*−/−*^ eyes corresponding with severe optic nerve damage (Fig. 7c). These results indicate that complete disruption of *Jnk2* and *Jnk3* is not sufficient to prevent JUN activation in RGCs or RGC death after an ocular hypertensive insult.

## Discussion

The stress-activated JNK signaling pathway promotes neurodegeneration in many systems and, thus, is widely considered an attractive target for neuroprotective therapies. The JNK pathway is known to promote axon degeneration and cause RGC death in settings with acute axon injury^13–18^, but was not tested in an age-related, ocular hypertensive model of glaucoma. Here it is demonstrated that activation of JNK2 and JNK3 are not required for glaucomatous degeneration in D2 mice. Instead, in the absence of JNK2, or both JNK2 and 3, there is an increase in ocular hypertension-induced neurodegeneration. An important implication of these data is that in a complex natural disease, manipulating JNK2 has an impact on disease outcomes unrelated to its role in directly activating soma or axon degeneration pathways.

The similar localization of pJNK observed in the ONH after mechanical optic nerve injury^14^ and in glaucoma (Fig. 1b) is consistent with the hypothesis that axon injury activates JNK in glaucoma. The importance of JNK signaling in RGC death in glaucoma and after optic nerve injury is supported by the increased RGC survival seen in these same two experimental models in *Jun-*deficient mice^13,15^. Thus, it is surprising that combined *Jnk2* and *3* deficiencies did not protect RGCs from death in glaucoma as it did after mechanical optic nerve injury. Our analyses of JNK signaling support the idea that differences occur at the level of JUN activation. Whereas JUN activation appears to be dependent on *Jnk2* and *3* after mechanical optic nerve injury^13^, JUN activation due to ocular hypertension in D2 mice still occurred in RGC somas in the absence of JNK2 and 3 (Fig. 7a). The antibody used in this study detected JUN phosphorylation at the serine 63 residue, a phosphorylation site that is primarily specific to the kinase activity of JNK1, JNK2, and JNK3 after insults^31^. These data show that JNK2 and 3 are not required for JUN activation within RGCs, and they suggest a role for JNK1 in glaucoma. It is unclear if the involvement of JNK1 specific to D2 glaucoma is the result of the differing nature of the insult (progressive, chronic ocular hypertension compared with acute and sudden mechanical axonal injury), the potential for ocular hypertension to directly perturb pathways in more than just RGC axons, the involvement of other cells besides RGCs, or the absence of JNK2 and 3. In future studies, it will be important to understand why the mechanism of JUN activation appears to differ between the contexts of a natural, age-related, progressive ocular hypertensive injury and an experimentally induced mechanical axonal injury. Nevertheless, these data highlight the importance of testing neuroprotective treatments in an age-related, chronic, ocular hypertensive model of glaucoma.

This work uncovered a novel neuroprotective function of JNK2 and provided an example of unequal contributions by JNK2 and JNK3 to glaucoma pathogenesis. Here, mice completely deficient in *Jnk2* progressed more quickly from onset of ocular hypertension to glaucomatous neurodegeneration than wild-type, *Jnk2* and *Jnk3* double heterozygous, or *Jnk3*-deficient mice. Although unlikely, as no heterozygous effects are reported, smaller effects associated with copy number of *Jnk2* and *Jnk3* cannot be dismissed due to the inclusion of *Jnk2* or *Jnk3* heterozygous mice in the study. Combining *Jnk2* and *Jnk3* deficiencies did have a small additional effect on optic nerve damage above the level of *Jnk2* deficiency. Overall, these data are consistent with JNK2 being a major mediator of neuroprotective JNK signaling after an ocular hypertensive injury. The prominent role of JNK2 may be related to a function of JNK2 that is not currently associated with other JNKs. Our data indicate that *Jnk2* was significantly downregulated in RGCs at a pre-degenerative stage of glaucoma in D2 mice (Fig. 1a). This early stage is marked by transcriptional responses related to metabolic and mitochondrial dysfunction within RGCs^29,32–34^. Of note, a unique function of JNK2 is to regulate mitophagy, in part through controlling degradation of smARF (a gene product of *Cdnk2b*) and indirectly p62^35^. Complete loss of JNK2 in RGCs may disrupt mitophagy early in glaucoma. Alternatively, JNK-deficient mice have altered immune responses^36–38^ and inflammatory responses in the nervous system^39,40^. Thus, disruption of *Jnk2* may affect how these types of responses contribute to glaucoma pathogenesis. In the experiments described here, mice carried a germline disruption of *Jnk2*. As the expression of *Jnk2* is ubiquitous in mammalian cells^41^, its absence may have affected the development, physiology, or stress response in any glaucoma-relevant cell type. Experiments that use cell type-specific disruption of *Jnk2* in RGCs and other glaucoma-relevant cells are necessary to further understand the role of JNK2 as a susceptibility factor for glaucoma.

In conclusion, this study provides further evidence implicating stress-activated kinases in the JNK family as modulators of RGC survival or death after axon injury and in glaucoma. The specific mechanisms that are impacted by the injury type and the final outcome is likely to be impacted by the competing pro-survival and pro-death effects of JNK activation. To effectively target JNK as a neuroprotective therapy, the pleiotropic effects of JNK isoforms need to be identified and specifically targeted.

## Materials and methods

### Mice

All experiments were conducted in accordance with the Association for Research in Vision and Ophthalmology statement on the use of animals in ophthalmic research and approved by The Jackson Laboratory Institutional Animal Care and Use Committee. Mice were housed with a 14-h light/10-h dark cycle and provided food and water ad libitum^42^. DBA/2J (D2), D2-*Gpnmb*^*+*^/J (D2-*Gpnmb*^*+*^), and mice carrying null alleles of *Jnk2 (Jnk2*^*-*^*; D2.129(B6)-Mapk9*^*<tm1Flv>*^*/*SjJ*)* and *Jnk3 (Jnk3*^*-*^*; D2.129S1(B6)-Mapk10*^*<tm1Flv>*^*/*SjJ*)* backcrossed to D2 for 13 generations have been previously described^4,13,24,43^. D2-*Gpnmb*^*+*^ mice are a substrain of D2 mice that have normal IOP at all ages and do not develop glaucoma^44^. Experimental cohorts of mice were generated using intercrosses of D2.*Jnk2*^*+/-*^ *Jnk3*^*+/-*^ (double heterozygous) mice. Mice referred to as control D2 mice include mice with wild-type (^+/+^) and heterozygous (^+/-^) genotypes for *Jnk2* and *Jnk3* (e.g., *Jnk2*^*+/+*^*Jnk3*^*+/+*^, *Jnk2*^*+/+*^*Jnk3*^*+/−*^, *Jnk2*^*+/−*^*Jnk3*^*+/+*^, and *Jnk2*^*+/−*^*Jnk3*^*+/−*^). Mice with genotypes of *Jnk2*^*−/−*^ include mice with *Jnk3*^*+/+*^ and *Jnk3*^*+/−*^ genotypes. Mice with genotypes of *Jnk3*^*−/−*^ include mice with *Jnk2*^*+/+*^ and *Jnk2*^*+/-*^ genotypes.

### Clinical exams and intraocular pressure measurement

D2 mice develop a form of pigmentary glaucoma. In D2 eyes, elevated IOP occurs secondary to a depigmenting iris disease^24,25,42^. Iris disease and intraocular pressure were evaluated in mutant mice and littermate control D2 mice using previously described methods^23,24,45^. Iris disease was assessed at 2-month intervals starting at 6 months of age until experimental completion in > 40 eyes of each genotype. Intraocular pressure was measured at 8.5, 10.0, and 12.0 months of age.

### Assessment of retina and optic nerve damage by histology

Mice were analyzed as three age groups: 9–10 mos, >10–11 mos, and 12–13 mos. Mice of each genotype within each age bin were age and sex matched. The method of preparing, staining, and analyzing plastic sections of retinas and optic nerves for damage has been previously described and validated^4,8,25,30,46,47^. Retinal cross-sections were stained with hematoxylin and eosin (H&E). Cross-sections of optic nerve were stained with paraphenylenediamine (PPD), which stains all myelin sheaths, but darkly stains the axoplasm of injured or dying axons. Individual damaged axons are readily identified within a generally healthy nerve. Two investigators assigned one of three damages level to each nerve. The investigators were masked to age, genotype, and damage level assigned by the other investigator. In rare cases of disagreement, a third masked investigator acted as a tie-breaker. Nerves with no or early glaucoma (NOE) have no detectable glaucomatous damage and are indistinguishable from D2.*Gpnmb*^*+*^ nerves. Moderately (MOD) affected nerves had readily detectable degenerating axons (average 30% axon loss) as marked by darkly stained axoplasm; however, the majority of axons were still healthy. Severely (SEV) affected nerves have >50% axon loss and prominent gliosis. Axon numbers are significantly different between optic nerves of each damage level^4,8,25,30,46,47^. To be used for histology or RGC counts representing severe glaucoma, a retina had to be associated with an optic nerve judged to have >95% of axons lost (i.e., entirely gliotic with few remaining axons).

### Immunohistochemistry

Eyes were fixed in 4% paraformaldehyde and subsequently either cryoprotected in 30% sucrose and frozen or the retinas were dissected for use in flat mounts. For cryosection immunostaining, slides were blocked in 10% horse serum in 0.1% Triton X-100 in phosphate-buffered saline (PBS) (PBST) for 2 h, incubated in primary antibody solutions overnight, and secondary antibody solutions for 2 h. Primary antibodies (rabbit anti-phosphorylated pan-JNK, pJNK, Cell Signaling, #4668) were diluted in PBST containing 2% goat serum. Alexafluor-conjugated secondary antibodies (Invitrogen) were diluted in PBST (AF488 and AF568) and nuclei were counterstained with 4,6-diamidino-2-phenylindole (DAPI). For flat mount immunostaining, retinas were blocked in 0.3% Triton X-100 in PBS containing 10% goat serum for 3–4 h, incubated in primary antibody solutions overnight and secondary antibody solutions for 3–4 h. Primary antibodies (rabbit anti-p-cJUN, Abcam, #ab32385, mouse anti-β-tubulin, Covance, #MMS-435P) were diluted in 0.3% Triton X-100 in PBS, and Alexafluor-conjugated secondary antibodies (Invitrogen) diluted in PBST for 24 h at 4°C. Following antibody incubations and washes, the retinas were mounted on slides in aquamount, coverslipped, and sealed with nail polish. Images of slides were taken using a Zeiss AxioImager.

### RNA-sequencing dataset analysis

The RGC transcriptome data used was from publically available RNA-sequencing data sets generated by our lab: GEO accession number GSE90654^29^. Differential gene expression analysis was performed in edgeR v3.10.5^48^ and plotted using custom R scripts.

### Statistical analyses

For IOP measurements, 40 eyes per genotype were assessed at each time point. Statistical analysis was performed using Student’s *t*-test and *Holm*-corrected *P*-values with *P* < 0.01 considered significant. Optic nerve disease stage profiles were compared between genotypes using Fisher exact and *Holm*-corrected *P*-values with *P* < 0.01 considered significant. Differential expression analysis of RNA-seq transcripts was adjusted for multiple testing using a FDR (*q*). Genes were considered to be significantly differentially expression at an FDR < 0.05.
